# Loneliness severity and heart disease risk in older Chinese rural adults: a machine learning-based cross-sectional study

**DOI:** 10.7189/jogh.15.04302

**Published:** 2025-10-17

**Authors:** Di Ding, Jiasheng Cai, Xusen Sun, Rong Luo, Haibo Liu

**Affiliations:** Department of Cardiology, Qingpu Branch of Zhongshan Hospital Affiliated to Fudan University, Shanghai, China

## Abstract

**Background:**

Loneliness is an important psychosocial determinant of cardiovascular health, yet its role in heart disease among older adults in rural China is underexplored.

**Methods:**

Using 2020 China Health and Retirement Longitudinal Study (CHARLS) data, the analytic sample comprised 5767 rural residents aged ≥60 years. Loneliness was measured with a four-level 10-item Center for Epidemiologic Studies Depression Scale (CESD-10) item. Covariate-adjusted, residence-stratified logistic regressions assessed associations. Six machine learning classifiers (including logistic regression and gradient boosting decision tree) were trained with a stratified 70/30 split and evaluated by area under the receiver operating characteristic curve (ROC-AUC), area under the precision–recall curve (PR-AUC), accuracy, precision, recall, and F1; decision-curve analysis quantified net benefit. SHapley Additive exPlanations (SHAP) provided model interpretability.

**Results:**

A graded association was observed: higher loneliness severity corresponded to higher odds of heart disease, with stronger effects in rural than urban strata. Logistic regression and gradient boosting decision tree showed the best discrimination (ROC-AUC 0.753 and 0.750; PR-AUC 0.400 and 0.388). Decision-curve analysis indicated greater net benefit for these models across clinically relevant thresholds. SHAP ranked dyslipidemia, sex, age, hypertension, and diabetes as leading contributors, and identified loneliness as an independent, quantifiable predictor.

**Conclusions:**

Loneliness is a salient correlate of self-reported heart disease in rural Chinese older adults. Incorporating brief loneliness screening into routine primary care may aid risk stratification in under-resourced settings. Interpretation should consider the cross-sectional design and self-reported outcomes; longitudinal studies with external validation and clinically adjudicated endpoints are warranted.

Cardiovascular diseases (CVD), particularly heart disease, remain a leading global health concern; the number of affected people has nearly doubled from 271 million in 1990 to 523 million in 2019 [1]. This rise is largely attributable to population ageing, a major driver of cardiovascular events across age strata [2,3]. In China, CVD affects approximately 330 million individuals and is the leading cause of mortality [4,5]. Together, these figures point to a growing absolute number of older adults living with heart disease and sustained pressure on prevention and care.

Numerous cross-national studies, including those from the USA, Canada, England & Wales, Germany and China, have revealed persistent urban–rural disparities in health outcomes [6-11]. These structural disadvantages, including lower resource availability, longer pre-hospital delays and limited access to specialist services, are directly pertinent to heart disease care and outcomes in older rural populations. In practice, such constraints may delay first contact and limit secondary prevention and continuity of care, which may help explain poorer control and late-life outcomes in rural communities.

Beyond biological and behavioural determinants, loneliness, defined as a subjective sense of social disconnection, has been linked to adverse cardiovascular outcomes, including coronary events and other forms of heart disease, and to all-cause mortality [12-17]. Proposed pathways include hypothalamic-pituitary-adrenal axis dysregulation, chronic low-grade inflammation, maladaptive health behaviours and poorer adherence [18]. These vulnerabilities are often amplified in older adults as social networks shrink with retirement, bereavement or functional decline [19,20]. Together, these mechanisms provide plausible channels through which loneliness may affect the recognition and management of heart disease, especially where social and clinical support are limited.

Evidence specific to older rural Chinese adults remains limited, despite indications of higher loneliness and disproportionate cardiovascular burden in this group [21,22]. In parallel, conventional clinical frameworks seldom incorporate psychosocial metrics, which may reduce discrimination in socially vulnerable populations [23-27]. Clarifying the role of loneliness in this population may offer a simple and scalable input for risk stratification and follow-up in primary care. Against this backdrop, the present study examines whether loneliness severity is associated with self-reported physician-diagnosed heart disease among rural Chinese adults aged ≥60 years and whether including loneliness improves discrimination for prevalent heart disease using conventional and machine-learning models.

## METHODS

### Participants

The analysis used data from the 2020 round (Wave 5) of the China Health and Retirement Longitudinal Study (CHARLS), a nationally representative longitudinal survey of Chinese residents aged ≥45 years [28,29]. Ethics approval was granted by the Peking University Biomedical Ethics Review Committee (IRB00001052-11015). Participants aged ≥60 years were eligible, and records with missing self-reported heart disease status or incomplete loneliness responses were excluded. Before restricting by residence, a descriptive contextual comparison by residential setting (urban *vs*. rural) was conducted in the eligible ≥60-year cohort. For the primary analysis, the sample was restricted to rural residents; urban residents were excluded from model development, performance evaluation, and interpretability analyses. After applying these criteria, 5767 rural respondents remained.

### Variable definitions

The primary health outcome was heart disease. Following the CHARLS protocol, trained interviewers asked participants in the 2020 survey: ‘Has a doctor ever told you that you have heart disease, such as myocardial infarction, coronary heart disease, angina, congestive heart failure, or other heart problems?’ Participants answering ‘yes’ were classified as having heart disease; all others were classified as not having heart disease [28,29]. The central independent variable was loneliness, derived from a single item on the 10-item Center for Epidemiologic Studies Depression Scale (CESD-10) scale that asked: ‘During the past week, how often did you feel lonely?’ Participants responded using four options:

1) rarely or never (less than one day);

2) sometimes (1–2 days);

3) occasionally (around three days); and

4) frequently (5–7 days) [30,31].

This variable was treated as an ordinal predictor and was entered directly into all analytical models. The control variables encompassed demographic characteristics (age as a continuous measure, sex as male or female) and health-related behaviours, including current smoking and drinking status (yes/no) [32,33]. Self-reported histories of hypertension, diabetes, dyslipidemia, and stroke were included as binary covariates [34]. As described in statistical analysis, preprocessing (standardisation) was fitted on the training folds only and applied across cross-validation folds and the test set to prevent data leakage.

### Machine learning models and evaluation

To explore the predictive utility of loneliness in identifying heart disease among rural older adults, six widely applied machine learning (ML) algorithms were developed and compared: logistic regression (LR), support vector machine (SVM), K-nearest neighbours (KNN), gradient boosting decision tree (GBDT), random forest (RF), and eXtreme Gradient Boosting (XGBoost). Model construction was performed in Python 3.10 (Python Software Foundation, Wilmington, DE, USA) using the scikit-learn and XGBoost libraries.

The data set was partitioned via a stratified random split into a training cohort (70%) and a testing cohort (30%). Within the training subset, 5-fold cross-validation coupled with grid search was used for hyperparameter tuning. The optimal configurations were then retrained on the full training data, and performance was evaluated on the held-out test set. Class imbalance was addressed using class weights. Discrimination and classification performance were assessed using area under the receiver operating characteristic curve (ROC-AUC), area under the precision-recall curve (PR-AUC), accuracy, precision, recall, and F1; the baseline precision for PR-AUC is the prevalence of heart disease in the evaluation set. Clinical applicability was assessed using decision curve analysis (DCA) to quantify net benefit across threshold probabilities of 0.05–0.50, with ‘treat none’ and ‘treat all’ as reference strategies.

For interpretability, SHapley Additive exPlanations (SHAP) values were calculated to quantify the marginal contribution of each input feature to individual predictions. The two best-performing models by test-set ROC-AUC were further examined using SHAP beeswarm plots and bar plots of mean absolute SHA*P* values. All figures were generated with Matplotlib 3.7.0 (Matplotlib Development Team/NumFOCUS, Austin, TX, USA) and Seaborn 0.13.2 (seaborn project; Michael L. Waskom, New York University, New York, NY, USA).

### Descriptive analysis and software environment

All preprocessing, descriptive statistics, model training, and figure generation were performed in Python 3.10 within a dedicated conda environment. Data handling used pandas; group comparisons and inferential tests (*e.g*. χ^2^, *t* test/analysis of variance (ANOVA) as appropriate) used SciPy. Stratified logistic regressions for residence-specific odds ratios were estimated with statsmodels. Machine-learning classifiers (logistic regression, SVM, K-nearest neighbours, gradient boosting decision tree, random forest, and XGBoost) were implemented with scikit-learn and XGBoost. Model interpretability used SHAP. Visualisations were produced with Matplotlib (v3.7.0) and Seaborn (v0.13.2). A consistent random seed was applied to data partitioning and model fitting to enhance reproducibility.

### Model interpretation using SHAP

To enhance interpretability and quantify each predictor’s contribution, SHA*P* values were computed for the top-performing classifiers. As a model-agnostic, game-theoretic framework, SHAP estimates the marginal influence of each input feature on individual predictions, providing both global and local explanations. In this analysis, SHAP was applied to the two classifiers with the highest test-set ROC-AUC (logistic regression and gradient boosting decision tree). Beeswarm plots summarised the distribution of SHA*P* values across observations, mean absolute SHAP value bar plots were used to derive aggregated feature rankings. These results elucidate how loneliness and other covariates contribute to the predicted probability of heart disease among older rural adults. The overall data-processing and modeling workflow is shown in Figure S1 in the **Online Supplementary Document**. All SHAP computations and visualisations were generated using the SHAP Python package, v0.45.0 (SHAP project, University of Washington, Seattle, WA, USA) with interoperability for scikit-learn and XGBoost model objects.

## RESULTS

### Population characteristics

A total of 5767 older adults residing in rural areas and 60 years or older were included in the final analytical sample. According to responses to the CESD-10 loneliness item, 3983 people (69.0%) reported no feelings of loneliness, 516 (8.9%) experienced light loneliness, 687 (11.9%) reported moderate loneliness, and 581 (10.1%) reported severe loneliness.

**Table 1** presents the baseline characteristics stratified by severity of loneliness. Individuals who reported greater loneliness were generally older (mean age 69.71 years in the severe group *vs*. 68.07 years in the no-loneliness group, *P* < 0.001) and more likely to be female (52.3% *vs*. 47.7%, *P* < 0.001). Smoking and alcohol consumption decreased with increasing severity of loneliness (smoking: 22.0% in the severe group *vs*. 27.4% in the no-loneliness group, *P* = 0.003; drinking: 25.8% *vs*. 34.9%, *P* < 0.001). Regarding comorbidities, individuals experiencing higher levels of loneliness exhibited significantly elevated rates of hypertension, diabetes, stroke, and dyslipidemia (all *P* < 0.05), suggesting a concentration of cardiometabolic burden within this subgroup.

**Table 1:** Baseline characteristics of rural elderly participants across loneliness severity levels

Variable name	No loneliness	Light	Moderate	Severe	*P*-value*
Age, x̄ (SD)	68.07 (6.20)	68.22 (6.19)	68.41 (6.29)	69.71 (6.40)	*<*0.001
Gender, n (%)					
*Male*	2083 (52.3)	253 (49.0)	274 (39.9)	214 (36.8)	*<*0.001
*Female*	1900 (47.7)	263 (51.0)	413 (60.1)	367 (63.2)	
Smoking status, n (%)					
*No*	2893 (72.6)	401 (77.7)	522 (76.0)	453 (78.0)	
*Yes*	1090 (27.4)	115 (22.3)	165 (24.0)	128 (22.0)	0.003
Drinking status, n (%)					
*No*	2592 (65.1)	361 (70.0)	496 (72.2)	431 (74.2)	<0.001
*Yes*	1391 (34.9)	155 (30.0)	191 (27.8)	150 (25.8)	
High blood pressure, n (%)					
*No*	2353 (59.1)	270 (52.3)	397 (57.8)	307 (52.8)	0.002
*Yes*	1630 (40.9)	246 (47.7)	290 (42.2)	274 (47.2)	
Diabetes, n (%)					
*No*	3465 (87.0)	430 (83.3)	593 (86.3)	480 (82.6)	0.008
*Yes*	518 (13.0)	86 (16.7)	94 (13.7)	101 (17.4)	
Stroke, n (%)					
*No*	3680 (92.4)	476 (92.2)	618 (90.0)	516 (88.8)	0.008
*Yes*	303 (7.6)	40 (7.8)	69 (10.0)	65 (11.2)	
Dyslipidemia, n (%)					
*No*	3079 (77.3)	402 (77.9)	501 (72.9)	431 (74.2)	0.035
*Yes*	904 (22.7)	114 (22.1)	186 (27.1)	150 (25.8)	

SD – standard deviation, x̄ – mean

*P-values were generated using variance analyses or χ^2^ tests.

**Figure 1:**
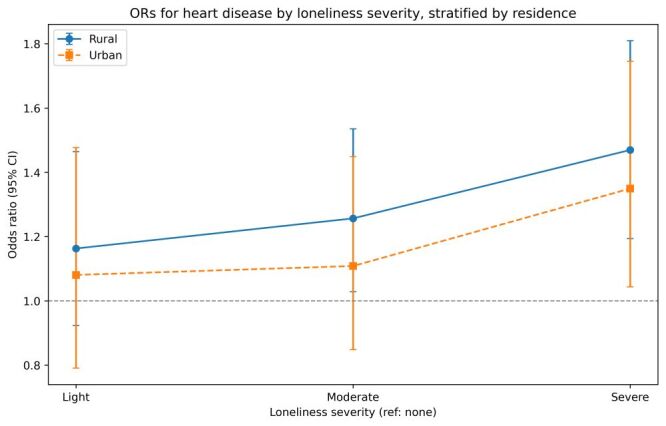
Odds ratios (ORs) for heart disease by loneliness severity from covariate-adjusted logistic models stratified by residence (rural *vs*. urban); reference = no loneliness. Adjusted for age, sex, current smoking, current drinking, hypertension (HTN), diabetes mellitus (DM), dyslipidemia, and stroke. Points denote ORs; error bars denote 95% confidence intervals.

To assess the differential associations of loneliness with heart disease across residential settings, stratified, covariate-adjusted logistic regression models were conducted. Increases in loneliness severity were associated with progressively higher odds of heart disease in both rural and urban populations (**Figure 1**). The association was more pronounced among rural participants, with odds ratios ranging from 1.16 (light) to 1.47 (severe), compared with 1.08 to 1.35 in urban counterparts. These results support the central hypothesis and underscore the increased vulnerability of older adults in rural regions to psychosocial risk exposures.

### Model performance comparison

**Figure 2:**
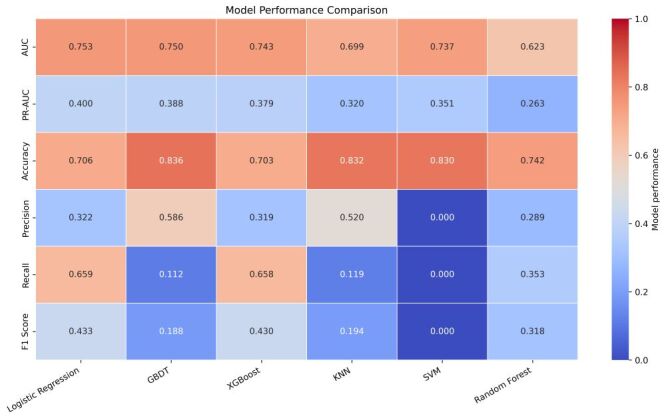
Model performance comparison (heatmap) showing area under the receiver operating characteristic curve (ROC-AUC), area under the precision-recall curve (PR-AUC), accuracy, precision, recall, and F1 on the held-out test set.

In the rural analytic sample, the prevalence of self-reported heart disease was 17.0%, which serves as the baseline precision for PR-AUC. Six ML classifiers (LR, GBDT, XGBoost, KNN, SVM, RF) were trained and evaluated using ROC-AUC, PR-AUC, accuracy, precision, recall, and F1. **Table 2** and **Figure 2** summarise performance. Logistic regression and GBDT achieved the highest ROC-AUC (0.753 and 0.750, respectively). PR-AUC was greatest for LR (0.400), followed by GBDT (0.388) and XGBoost (0.379). Accuracy was highest for GBDT (0.836), with KNN (0.832) and SVM (0.830) close behind. Precision was highest for GBDT (0.586) and KNN (0.520). Recall was highest for LR (0.659) and XGBoost (0.658), yielding the top F1 scores for LR (0.433) and XGBoost (0.430). Random forest showed the lowest ROC-AUC (0.623) and PR-AUC (0.263). Although SVM reached a reasonable ROC-AUC (0.737), its precision, recall, and F1 were near zero at the default 0.5 threshold, indicating poor classification at that cut-off.

**Table 2:** Performance comparison of ML models for heart disease prediction

Models	ROC-AUC	PR-AUC	Precision	Accuracy	Recall	F1 Score
LR	0.753	0.400	0.322	0.706	0.659	0.433
GBDT	0.750	0.388	0.586	0.836	0.112	0.188
XGBoost	0.743	0.379	0.319	0.703	0.658	0.430
SVM	0.737	0.351	0	0.830	0	0
KNN	0.699	0.320	0.520	0.832	0.119	0.194
RF	0.623	0.263	0.289	0.742	0.353	0.318

GBDT – gradient boosting decision tree, KNN – k-nearest neighbours, LR – logistic regression, PR-AUC – area under the precision-recall curve, RF – random forest, ROC-AUC – area under the receiver operating characteristic curve, SVM – support vector machine, XGBoost – Extreme Gradient Boosting

**Figure 3:**
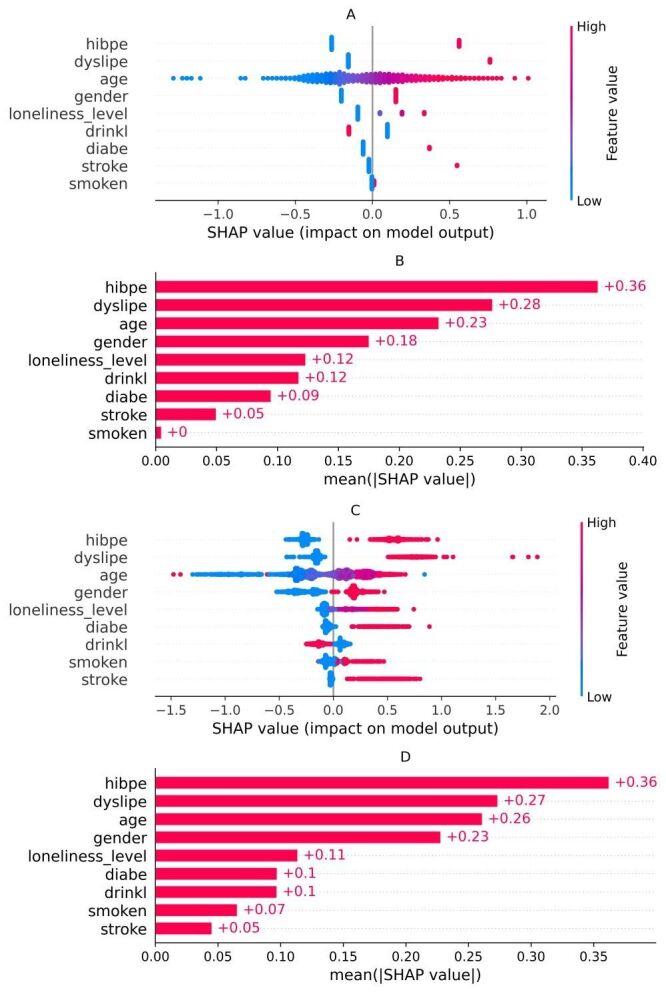
SHAP-based interpretability for the top two models (**Panel A** and** Panel B**: LR; **Panel C** and** Panel D**: GBDT). Beeswarm panels display the distribution and direction of SHAP values; bar panels show mean absolute SHAP values for aggregated feature importance.

Figure S2 in the **Online Supplementary Document** displays ROC curves that corroborate the superior discrimination of LR and GBDT across a broad range of false-positive rates. Figure S3 in the **Online Supplementary Document** shows precision-recall curves consistent with these patterns when interpreted against the 17.0% prevalence baseline. Figure S4 in the **Online Supplementary Document** presents decision-curve analysis, demonstrating greater net benefit for LR and GBDT than treat-none and treat-all strategies across most clinically relevant thresholds. On the basis of these complementary evaluations, LR and GBDT were retained for SHAP-based interpretability (**Figure 3**).

### Model interpretation and feature contribution analysis

To elucidate the decision mechanisms of the top-performing models, SHAP analysis was applied to LR and GBDT. The SHAP beeswarm panels (A/C) and the corresponding bar plots of mean absolute SHA*P* values (B/D) depict the relative importance and directional impact of each input variable on the predicted risk of heart disease (**Figure 3**). Among the most influential features, dyslipidemia had the largest mean absolute SHAP value, followed by sex, age, hypertension, and diabetes, consistent with established cardiovascular risk factors. Loneliness also contributed meaningfully: higher loneliness categories corresponded to larger positive SHA*P* values, indicating greater influence on the predicted probability of heart disease.

The beeswarm plots further show that participants reporting higher levels of loneliness (red points to the right of zero) tended to receive higher predicted probabilities, particularly when accompanied by other high-risk attributes such as older age and dyslipidemia. These findings highlight the additive, context-dependent contribution of loneliness as a psychosocial determinant within the modeling framework.

Taken together, the results support including loneliness severity as an independent, quantifiable predictor in risk-stratification approaches for rural older adults.

## DISCUSSION

This study builds on growing evidence that psychosocial influences, especially loneliness, have a tangible impact on cardiovascular outcomes, particularly in populations facing structural disadvantages. By incorporating loneliness severity into machine-learning models alongside standard clinical predictors, the analysis indicates that loneliness is independently linked to heart disease among older adults in rural China and likely interacts with environmental and social disadvantages to amplify risk. These findings challenge biomedical perspectives that overlook lived experience, underscoring the need to consider social context when evaluating risk in under-resourced settings [4,5]. In the test set, discrimination was moderate (ROC-AUC ~ 0.75 for the leading models), and PR-AUC exceeded the baseline prevalence ( ~ 0.17), indicating added value under class-imbalance conditions.

Although prior work has described biological pathways connecting loneliness with hypothalamic-pituitary-adrenal-axis dysregulation, chronic low-grade inflammation, and adverse behaviours such as reduced physical activity, disrupted sleep, and tobacco use [17,18], its value as a predictive factor in rural cardiovascular health has received limited attention. In this analysis, loneliness, measured on a four-level scale, emerged as a non-overlapping predictor of self-reported disease, retaining importance after adjustment for hypertension, dyslipidemia, diabetes, and other comorbidities. SHAP-based interpretation further suggested that the predictive weight of loneliness approached that of key clinical markers, with a more pronounced pattern among rural residents. This profile is consistent with settings where psychosocial stressors, limited medical infrastructure, social isolation, and technological barriers co-occur [6,8]. Complementing discrimination metrics, decision-curve analysis showed positive net benefit for the top models over treat-none and treat-all strategies across clinically plausible threshold probabilities (*e.g*. approximately 0.05–0.50), supporting potential screening utility in primary care.

These observations align with emerging evidence from China linking loneliness with poor perceived health, reduced functional capacity, and higher chronic-disease burden [34,35]. Earlier studies seldom focused explicitly on heart disease or used methods that capture nonlinearities and interactions. By applying multiple models and interpretability tools, the present work extends prior research with methodological breadth and local relevance. Unlike traditional regressions, the applied models can highlight subgroup-specific risk patterns and latent interdependencies [26]. Importantly, the consistency of findings across metrics (ROC-AUC, PR-AUC) and clinical utility (DCA) supports the consideration of psychosocial inputs in pragmatic risk workflows.

Integration of loneliness into risk modeling offers practical value for assessment and care among older adults in rural areas. Conventional cardiovascular risk tools derived from epidemiologic cohorts rarely incorporate psychosocial variables despite their recognised role in disease development [36]. The current results indicate that even a single-item loneliness indicator can enhance predictive performance and provide a low-cost signal for case finding in community settings. A feasible workflow is to embed a brief loneliness question during routine chronic-disease follow-up in village clinics; positive screens can prompt brief counseling and community referral, with re-assessment at 3–6-month intervals. In resource-constrained environments, this stepwise approach can be implemented by local health workers without substantial additional infrastructure [22].

From a broader perspective, population-level strategies to reduce cardiovascular burden should also address upstream social conditions that shape loneliness and related stressors. Programmes that foster social connection – such as elder peer-support groups, rural mental-health outreach, or digital engagement initiatives – may alleviate isolation and mitigate physiological consequences [12,20]. At the same time, structural challenges including underinvestment in rural health services, fragmented care delivery, and digital exclusion act as compounding factors that intensify biological risk and perpetuate disparities [11]. Targeted service strengthening, combined with routine psychosocial screening, may therefore yield incremental benefits in high-risk rural cohorts.

Several limitations warrant consideration. First, heart disease was ascertained by self-reported physician diagnosis without clinical adjudication, which may introduce recall or awareness bias; non-differential misclassification would likely attenuate associations toward the null, whereas differential error could bias in either direction. Second, residential context was defined at the 2020 survey without information on duration of rural residence or recent migration, raising the possibility of contextual exposure misclassification that could attenuate urban-rural contrasts. Third, the cross-sectional design with a prevalent outcome precludes causal inference and temporality between loneliness and heart disease. Fourth, loneliness was captured by a single CESD-10 item rather than a multidimensional instrument, which may introduce measurement error and attenuate effect sizes. Fifth, residual confounding by unmeasured social and clinical factors (*e.g*. income, access to care, depressive symptoms, lipid profiles) may remain. Finally, models were developed and evaluated within one survey round; external validation on independent data would strengthen generalisability.

## CONCLUSIONS

This study demonstrates that loneliness is a prominent, independent psychosocial correlate of self-reported heart disease among older adults in rural China. Using nationally representative 2020 CHARLS data and multiple machine-learning algorithms, the analysis shows a graded association between loneliness severity and higher odds of prevalent heart disease after adjustment for conventional clinical factors. SHAP-based interpretation further ranks loneliness among the most informative predictors, with particularly pronounced effects observed in rural residents.

These findings support incorporating psychosocial variables, such as loneliness, into heart disease risk-assessment frameworks, especially in structurally disadvantaged populations. Given the moderate discrimination of current models and the compound vulnerabilities faced by older rural adults, embedding brief loneliness assessments into routine care workflows may aid early identification of individuals at elevated risk. Future research should include longitudinal designs, more comprehensive psychosocial profiling, externally validated models, and clinically verified endpoints to refine prediction and inform equitable, context-sensitive interventions.

## Additional material


Online Supplementary Document
