# Supplementary material for: Loneliness severity and heart disease risk in older Chinese rural adults: a machine learning-based cross-sectional study
Source: J Glob Health. 2025 Oct 17;15:04302. doi: 10.7189/jogh.15.04302 (PMC12531889; doi:10.7189/jogh.15.04302)

Supplement to: Ding D, Cai J, Sun X, Luo R, Liu H. Loneliness severity and heart disease risk in older Chinese rural adults: a machine learning-based cross-sectional study. J Glob Health. 2025;15:04302.

Figure S1. Workflow.

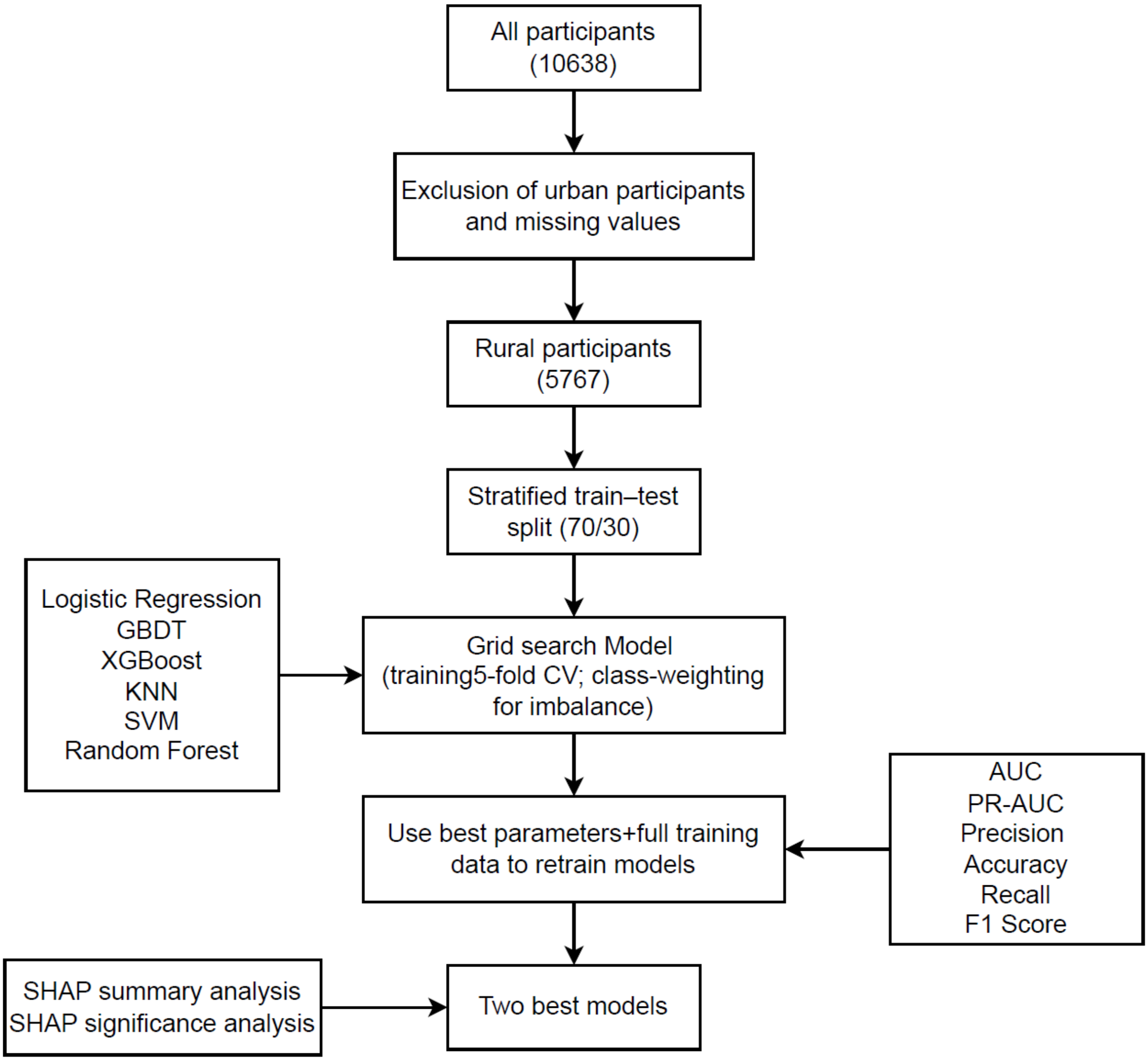

Figure S2. ROC curves of six clasifiers.

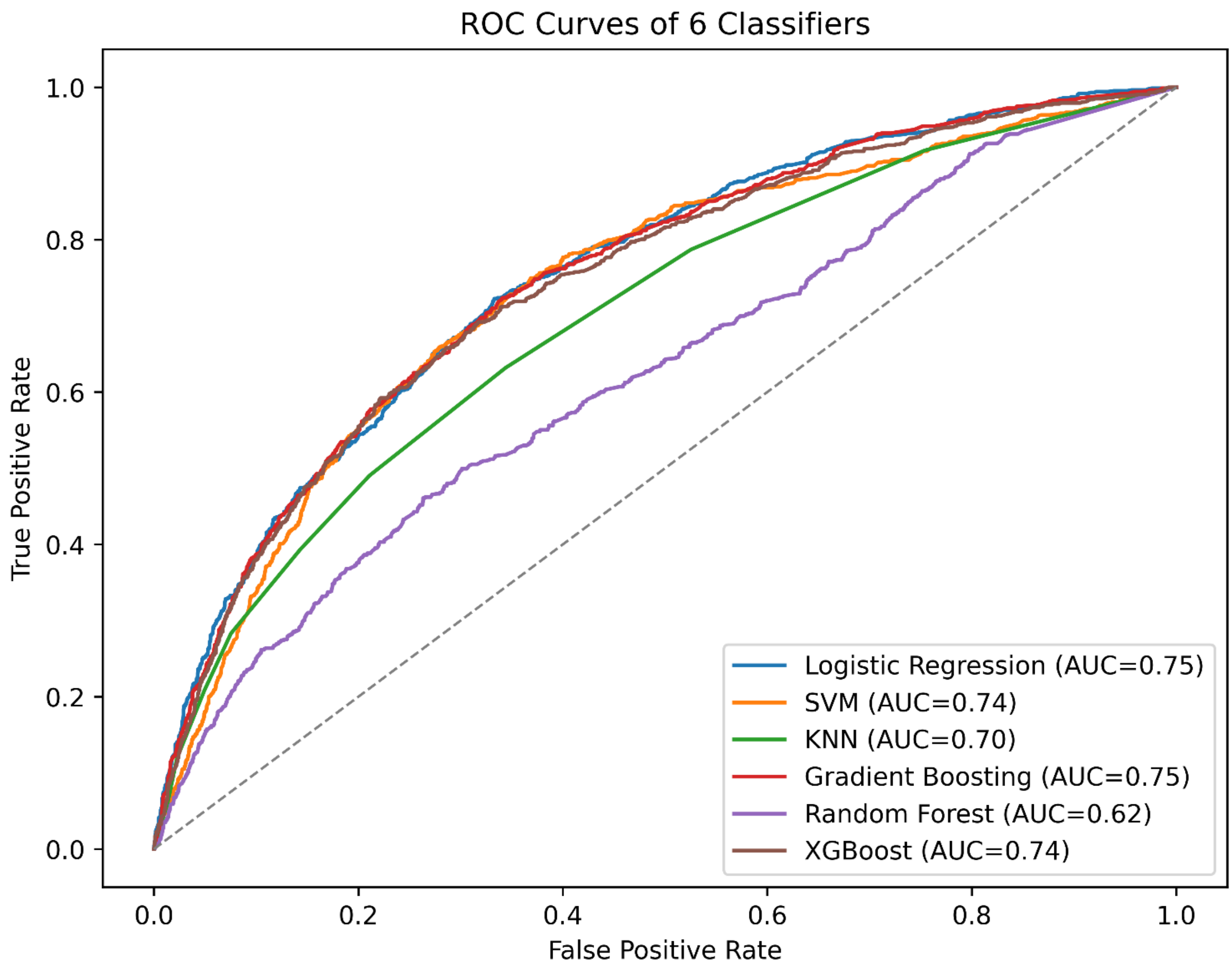

Figure S3. Precision recall curves.

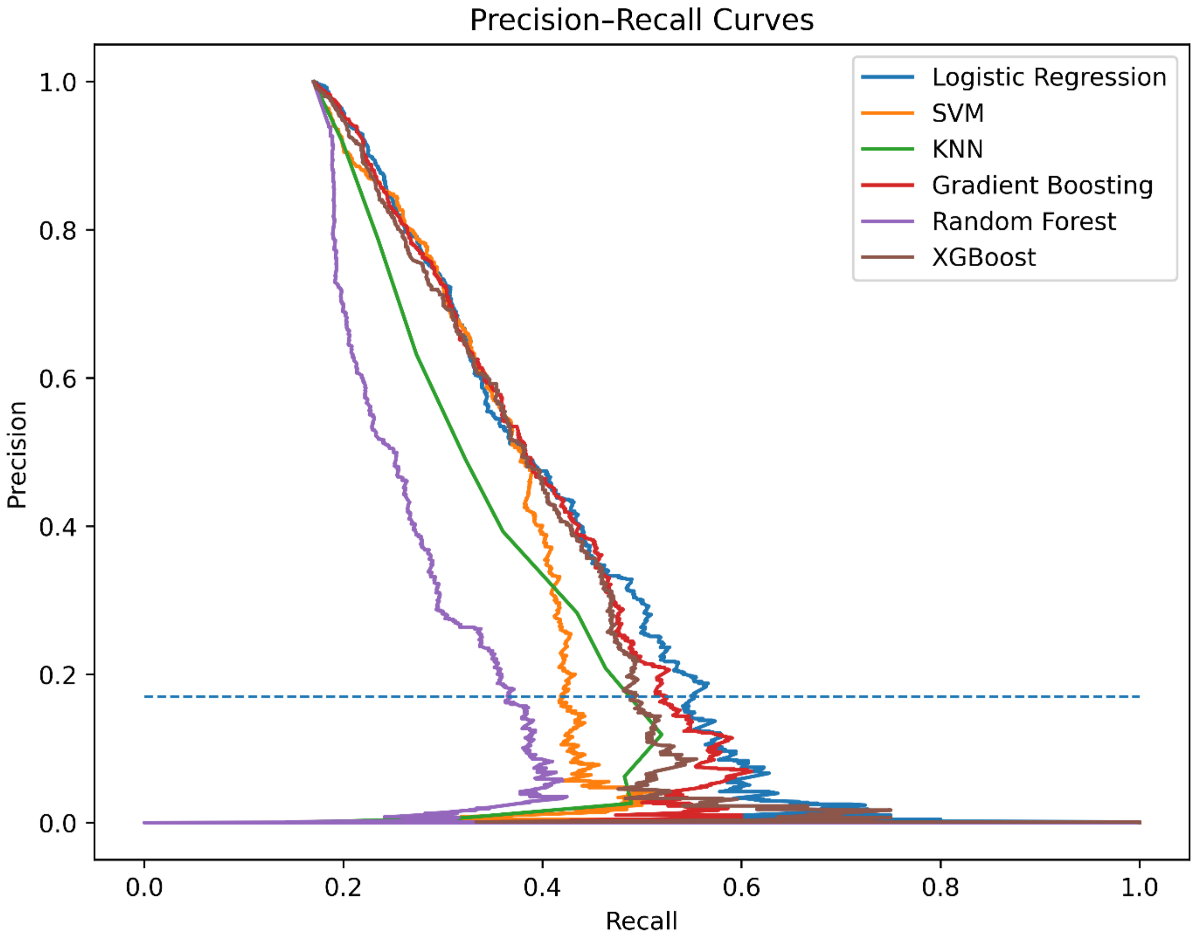

Figure S4. Decision curve analysis for heart disease risk prediction

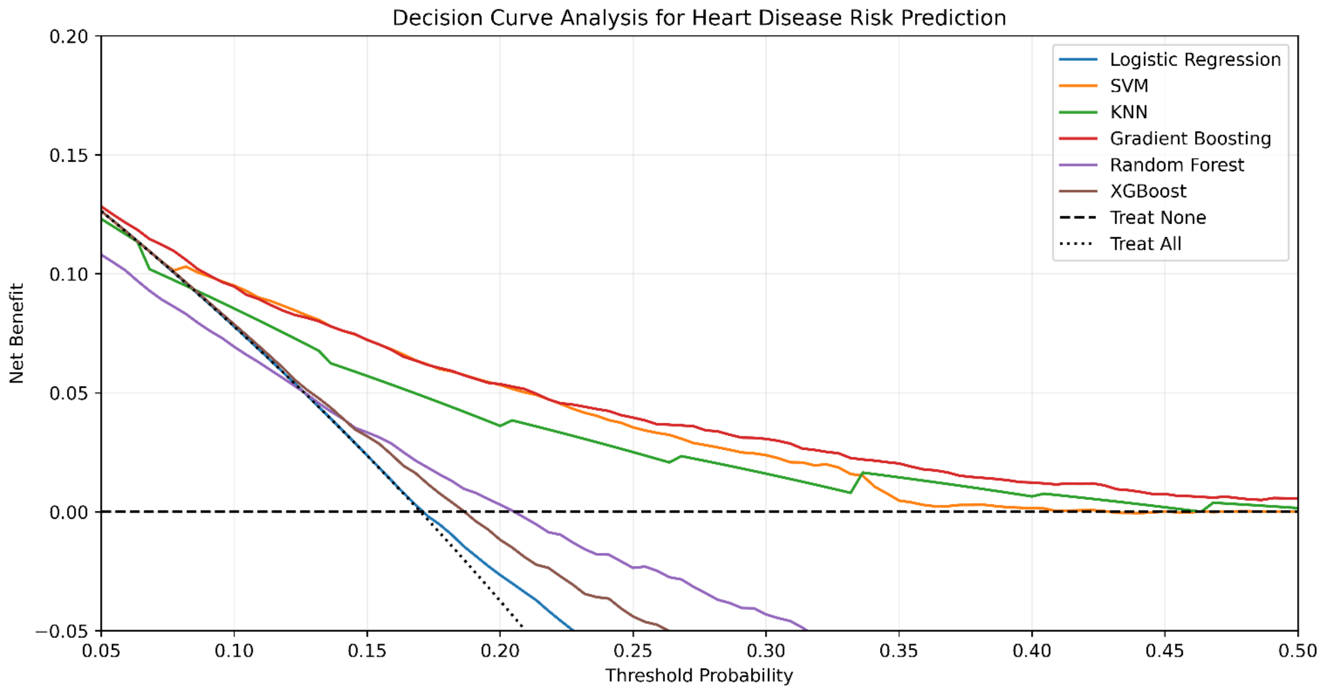

Supplement: Online Supplementary Document [file jogh-15-04302-s001.pdf]
